# Electropolishing and Shaping of Micro-Scale Metallic Features

**DOI:** 10.3390/mi13030468

**Published:** 2022-03-18

**Authors:** Sana Zaki, Nan Zhang, Michael D. Gilchrist

**Affiliations:** Center of Micro/Nano Manufacturing Technology (MNMT-Dublin), School of Mechanical & Materials Engineering, University College Dublin, Belfield, D04 V1W8 Dublin, Ireland; sana.zaki@ucdconnect.ie

**Keywords:** electropolishing, micro-fabrication, shaping, forming

## Abstract

Electropolishing (EP) is most widely used as a metal finishing process. It is a non-contact electrochemical process that can clean, passivate, deburr, brighten, and improve the biocompatibility of surfaces. However, there is clear potential for it to be used to shape and form the topology of micro-scale surface features, such as those found on the micro-applications of additively manufactured (AM) parts, transmission electron microscopy (TEM) samples, micro-electromechanical systems (MEMs), biomedical stents, and artificial implants. This review focuses on the fundamental principles of electrochemical polishing, the associated process parameters (voltage, current density, electrolytes, electrode gap, and time), and the increasing demand for using environmentally sustainable electrolytes and micro-scale applications. A summary of other micro-fabrication processes, including micro-milling, micro-electric discharge machining (EDM), laser polishing/ablation, lithography (LIGA), electrochemical etching (MacEtch), and reactive ion etching (RIE), are discussed and compared with EP. However, those processes have tool size, stress, wear, and structural integrity limitations for micro-structures. Hence, electropolishing offers two-fold benefits of material removal from the metal, resulting in a smooth and bright surface, along with the ability to shape/form micro-scale features, which makes the process particularly attractive for precision engineering applications.zx3.

## 1. Introduction

### 1.1. Overview

**Figure 1:**
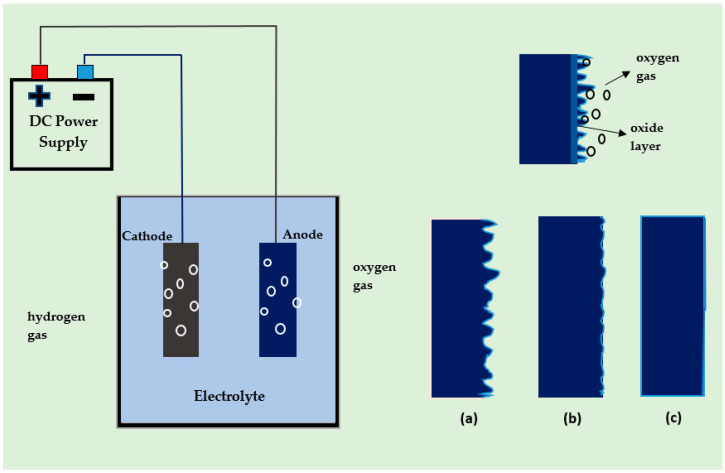
The basic setup of an electropolishing cell, showing how the surface of a workpiece changes from being initially uneven to being smooth, (**a**) Initial uneven surface of the workpiece, (**b**) Macro-levelling/finishing, (**c**) Micro-levelling/finishing (after Lee 2000 and Wang 2016 [18,19]).

Electropolishing is an electrolytic process for brightening, smoothening, and deburring metals. It levels the surface of a metal by removing material through an electrochemical process that is similar to, but the reverse of, electroplating [1,2]. A primary electropolishing cell in Figure 1 shows a progressive change in the surface of a workpiece from being uneven to being smooth. This is achieved after macro-polishing the surface, i.e., removing large-scale (~1 μm) irregularities and removing small-scale (~0.01 μm) irregularities via micro-polishing. Silver was the first metal to be electropolished using a cyanide solution patented by the German government [3,4]. At the same time, the theoretical basis for electropolishing was established in 1936 by Jacquet [5], who examined the electropolishing of copper in phosphoric acid. After processing, the metal is smoother and shinier, and it also achieves higher hardness, corrosion resistance, passivity, and bio-compatibility. Steel is the most commonly electropolished metal, due to its widespread use, ranging from macro-scale tools to micro-scale biomedical stents and nano-textured applications [2,6,7,8,9]. However, there is a growing demand for electropolished titanium, aluminum, niobium, copper, and nickel materials for applications that include superconducting channels, micro-electrochemical and bio-medical products [10,11,12,13,14,15,16,17]. Unlike the conventional finishing processes of grinding, milling, reaming, and buffing, ion by ion removal from the surface of metals is executed using electrolysis. Electropolishing is currently used in diverse industries, including medical devices, aerospace, automotive, petrochemical, food and beverages, and semiconductors.

### 1.2. Basic Principles

**Figure 2:**
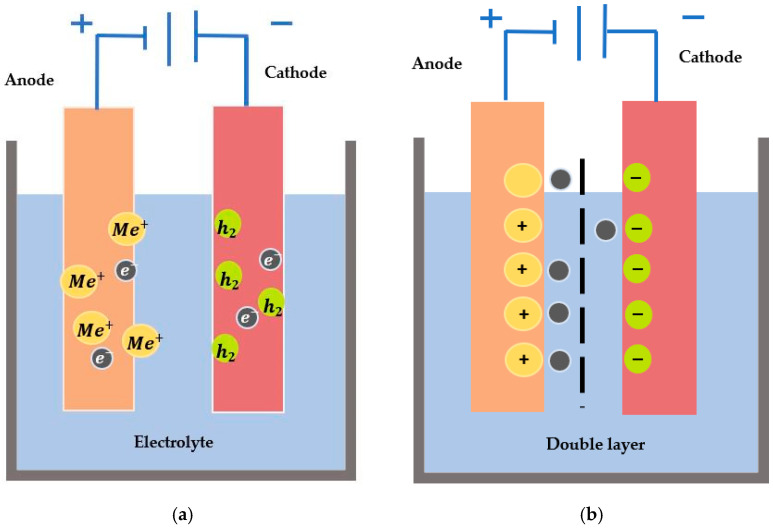
(**a**) Fundamental kinetics of electropolishing showing anodic dissolution and cathodic reduction, (**b**) The formation of the double layer when electrodes are immersed in an electrolyte and move across the barrier.

The metal intended to be electropolished acts as an anode, while the corresponding cathode is a counter electrode immersed in an appropriate electrolyte. As shown in Figure 2a, oxidation will occur at the anode, while hydrogen will be liberated at the cathode. The electrolyte needs to be a conductive medium for ions and metal dissolution. Conventionally, it is a strong acid, with some surfactants or additives being used to improve the electrodes’ viscosity, wettability, and reactivity. Sulfuric and phosphoric acids are used widely to electropolish metals and alloys [20,21,22,23,24,25,26,27,28,29]. Since acidic electrolytes can be hazardous or harmful to human safety and the environment, there is a growing trend of using green electrolytes. To this end, NaCl and mixtures of choline chloride and ethylene glycol have been investigated in modern studies [30,31,32,33,34,35,36,37,38,39,40,41]. 

The principles of electrochemistry are based on Faraday’s law from the 19th century [42]. These laws are related to the removal and dissolution rates of metal immersed in an electrolyte. It is an anodic treatment of the workpiece when immersed in an electrolytic solution [43]. When both electrodes are immersed in an electrolyte, a double layer is formed at the electrode interfaces (comprised of charges: one on the side of the electrode, and the other in the electrolyte), as highlighted in Figure 2b, and has been explained by various double layer models (e.g., Helmholtz (1879), Guy-Chapman (1910–1913), Stern (1924) and Grahame (1947)) that are related to the ionic orientation and rate of adsorption [44]. The critical factors that determine the level of surface polish include process time, voltage, current density, temperature, and electrolyte.

The amount of metal dissolution is governed by Faraday’s law, which is related to the amount of metal removed for a given current and time. This can be expressed in mass “*m*”, volume “*V*”, and height “*h*”, as per Equations (1)–(3), respectively [45].

(1) $$m=\frac{a}{\rho ZF}Q=\frac{a}{ZF}It\;\left(g\right)\;$$

(2) $$V=\frac{a}{\rho ZF}It\;\left({\mathrm{mm}}^{3}\right)\;$$

(3) $$h=\frac{a}{AZF}It\;\left(\mathrm{mm}\right)\;$$

where “*A*” is the surface area of a workpiece, “*Q*” the electric charge, “*a*” the atomic molecular weight, “*ρ*” the density, “*Z*” the valence of ions, and “*F*” the Faraday constant, “*I*” the current, and “*t*” the time.

**Figure 3:**
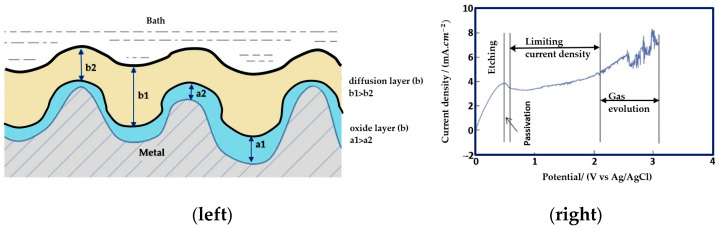
(**left**) Initial oxide and a diffusion layer on the metal surface [9], (**right**) Typical current–voltage characterisation curve (I–V) for electropolishing (four regions of etching, passivation, limiting current density, and gas evolution [46]. (Open access article distributed under the terms and conditions of the Creative Commons Attribution (CC BY) license).

An initial oxide layer forms on the surface of a metal when it is immersed in an electrolytic solution, as shown in Figure 3. Assume the metal’s surface is uneven, with a1, a2, b1 and b2 indicating the maximum and minimum thicknesses of the oxide and diffusion layers, respectively. The voltage application will result in higher current density at peak regions than at the valleys of the metal surfaces. The dissolution process starts with a gradient difference of oxide and diffusion layers that will be non-uniform and eventually result in a uniform oxide layer. To identify the current limiting plateau, a characterization of electrolytes is performed. It shows all four regions of etching, passivation, current limiting plateau, and gas evolution, as shown in Figure 3 (right). As there is no cutting tool involved, and material is removed via the movement of ions, this makes electropolishing a non-contact, atom-by-atom removal process to electrochemically polish and shape micro-scale features. Hence, electropolishing is helpful for the post-processing of additively manufactured (AM) parts, micro-electromechanical systems (radio frequency (RF MEMS or micro-optical electromechanical systems (MOEMS))), and transmission electron micro-scopy (TEM) sample preparation.

The concept of localized material removal results in better surface quality and finish. This is in contrast to other manufacturing processes, such as micro-milling, UV lithography, laser polishing, and chemical etching, none of which can provide the same accuracy in shaping micro-features, due to the limitations of tool size and wear, the rectilinear nature of light, and surface defects and stresses that are induced at tool–micro-feature interfaces [47,48,49,50,51,52]. Moreover, electrochemical polishing is capable of removing material at controlled rates using various waveforms: pulsed current (PC) and pulse reverse pulse current (PPR) can help maintain the structural integrity of micro-scale features [53,54,55,56,57]. The critical applications for the electrochemical polishing of micro-structures include TEM sample preparation [29,58,59], AM parts [12,60,61,62], biomedical stents and implants [7,17,54,56,63], and RF MEMS or MOEMS [64,65,66,67,68], which will be explained later in this review. There is also potential for electropolishing to be used as a shaping and forming process in the manufacturing of micro-mold tool inserts in order to boost the performance of micro-injection molding by preventing demolding problems.

This review highlights the potential of electrochemical polishing to both polish and shape the topology of micro-scale features. Some of the recent studies of electropolishing have focussed on fundamental theories, metal/alloy applications, microscopic technologies to evaluate electropolished surfaces, plasma electrolytic polishing (PEP), and magneto electropolishing (MEP) [69,70,71,72]. However, the current paper includes a brief comparative review of conventional and newer micro-machining techniques and their limitations, along with the theoretical background for electropolishing and a review of the optimization of process parameters and the micro-scale applications of electropolishing.

## 2. Alternative Micro-Feature Fabrication Processes

### 2.1. Micro-Milling

**Figure 4:**
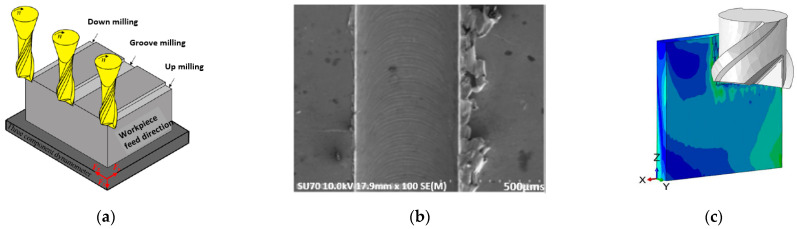
(**a**) Schematic of micro-channel machining [47], (**b**) formation of burrs [76] and (**c**) deflection of micro-tool [75]. Copyright granted by Elsevier and Optica Publishing Group.

Many miniaturized components can be manufactured through micro-milling. It usually involves cutting tools of very small diameter, typically 25 μm or 50 μm, and a high length-to-diameter ratio. A purpose-designed tool can remove material from the workpiece mechanically through micro-channels. Mechanical micro-machining is capable of achieving unique features and small tolerances with various materials, including hardened steel, Inconel, aluminum, and polymer composites [47,48,73,74]. However, there are real-time challenges of chip removal in micro-sizes, tool deformation, burr formation, and vibrations [47,75,76]. Figure 4 shows some of the micro-milling procedures: down-, groove-, and up-milling, burr formation, and the problem of tool deflection while milling thin-walled structures. These issues adversely affect the surface quality and finish of micro-features. Moreover, the removal of such a drill from a workpiece can damage the structure and cause unwanted deformation. Hence, micro-milling is not always a feasible or effective option for making micro-mold tools with certain features.

### 2.2. Micro-EDM

**Figure 5:**
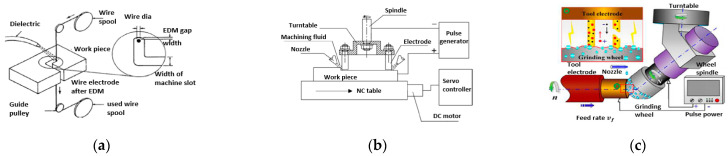
Different forms of electric discharge machining (EDM), (**a**) Wire EDM [82], (**b**) Electric discharge milling [83], (**c**) Electric grinding [84]. Copyright granted by Elsevier.

Electric discharge machining (EDM) is an advanced form of machining which achieves intricate shapes with good dimensional accuracy and surface finish, without the need to use specially designed electrodes [77]. Essentially, a beam of electrons travels from a cathode to an anode in the presence of a suitable voltage when both are immersed in a dielectric medium. The workpiece is melted and vaporized by means of a plasma. Eroded material is in the form of debris and is removed with each passing voltage cycle. EDM is a non-contact machining process that is suitable for high hardness and high strength materials with no issues of mechanical vibration; the distance between tool and workpiece is 20 to 40 um [78]. Some of the electric discharge machining forms are shown in Figure 5, namely, wire cutting, milling, and grinding. Wire EDM uses a thin wire as a tool to cut away material controlled by CNC, while electric discharge milling uses a rotating tool that follows a CNC code specified path, and electric grinding uses a conductive wheel as a tool to remove material. Recent advances have seen micro-EDM tools being made of hot-pressed copper composites to cut AISI D3 steel and its alloys, Inconel and slicing of silicon wafers <200 μm [78,79,80,81]. Despite the ability to cut very hard materials with complex shapes, there can be serious issues with pores, cracks, surface pits, and holes that affect morphology. Moreover, the operating cost and toxic environmental effects of μ-EDM mean that it can be an unattractive choice for micro-machining.

### 2.3. Laser Polishing/Ablation

**Figure 6:**
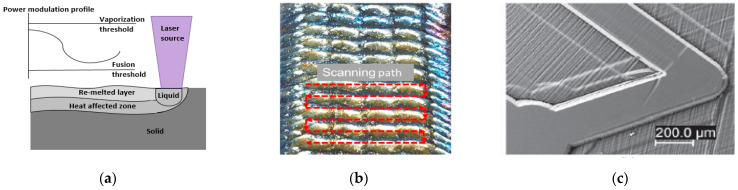
(**a**) Laser working principle (after Perry 2007) [85], (**b**) low and high power laser to remove material in additive manufactured AM part [90], (**c**) micro-feature on glassy carbon after laser polishing and cleaning [91]. Copyright granted by CCC and author (Kun Li 2021 [91]).

Laser technology has gained prominence in many areas of micro-machining. Lasers emit high beams of power, and the term LASER defines light amplification via the stimulated emission of radiation. The growth in the use of advanced materials like ceramics, composites, superalloys, and high-performance polymers increased the demand for this technology to machine complex shape parts with high levels of polish. The energy source is a laser beam that travels in a straight line and melts the area to be machined. Various zones are observed on the surface of a laser polished sample: heat-affected zone (HAZ), remelted layer, and liquid, as depicted in Figure 6a [85]. A laser-polished surface is characterized by the amount of material removed; the polishing that removes 10–80 μm of material by continuous remelting is referred to as macro-laser polishing [86,87,88], while remelting depths in the range of 0.5–5 μm are referred to as micro-laser polishing [88,89].

Lasers are used in diverse applications: cutting, welding, engraving, drilling, soldering, ablation, etching, and curing. Even micro-/nano-scale polishing has been achieved with laser polishing/ablation, resulting in high surface finish. Some of the recent research highlights the use of two step laser polishing on additive manufactured Ti_6_Al_4_V parts (Figure 6b), ultrasonic pulse applied to steel and laser polishing of glassy carbon (Figure 6c), and the use of Yb laser at ablation rates of 2.5~10 μm^3^/pulse [90,91,92]. Various laser parameters, such as low- and high-power beams, ablation rates, and the depth of melt pool, have been investigated in studies to machine features and improve the surface quality. Limitations of laser polishing are that it has particularly high initial costs, requires the use of a gas chamber, and can cause the oxidation and cracking of the surface metal. The rectilinear movement of a laser makes the shaping or forming of a feature around the corner of a workpiece difficult. This often limits its use for the micro-machining of features.

### 2.4. LIGA

**Figure 7:**
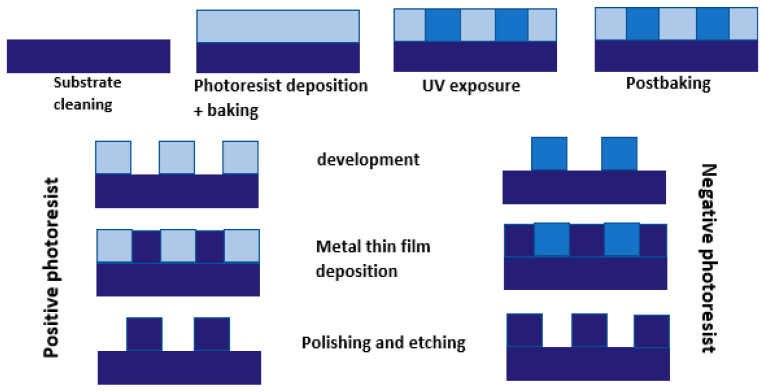
Basics steps of UV photolithography for micro-feature fabrication [101] (after Museau 2007 [101]).

LIGA has played a decisive role in fabricating integrated circuits since the 1990s. LIGA is a German abbreviation for Lithographie (lithography), Galvanoformung (electroforming), and Abformung (molding). Lithography, often referred to as photoengraving, is the process of transferring a pattern into a reactive polymer film (termed a resist), which is used subsequently to replicate that pattern into an underlying thin film or conductor [93]. This technology revolutionized the semi-conductor industry from planar transistors to high-aspect ratio features, i.e., ultra-large-scale integrated silicon (ULSI) circuits. LIGA is based on two fabrication technologies: X-ray and UV; the former uses X-rays to produce synchrotron, resulting in high-aspect ratio features, while the latter uses ultraviolet light to create low-aspect ratios by masking, and developing resist and metallic coatings. Figure 7 shows the basic steps of UV photolithography, which involve cleaning the substrate, depositing photoresist, its exposure to UV to generate positive or negative photoresist and, finally, electrodeposition. LIGA and its related processes have significant applications in manufacturing micro-pumps, gear wheels, micro-connectors, and biomedical inserts [94,95,96]. Lithography is used widely for the patterning of micro-channels and features by using double electroplating to make micro-gears, sacrificial layers for micro-motors, micro-needle arrays, and micro-fiducial markers for determining micro-mechanical properties [97,98,99,100]. Its advantages for micro-fabrication over electric discharge machining are that it offers high-aspect ratio features, and the high straightness and planarity of sidewalls. The limitations of the process include the high cost of the X-ray synchrotron source and the rectilinear nature of light, which makes it difficult to manufacture angled and formed geometries. Lithography has the capability to produce micro-patterns with good surface quality and aspect ratio, while electropolishing can enable the shaping of long channels based on localized material removal. So, a combined use of lithography and electropolishing can develop micro-channels or features that can be shaped and have a high surface finish.

### 2.5. Electrochemical Etching

**Figure 8:**
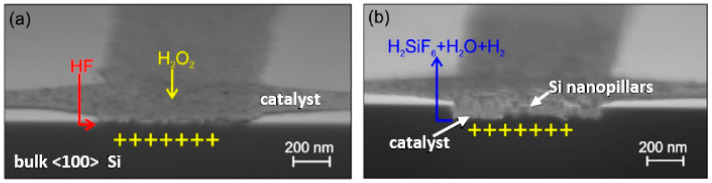
Process of MacEtch with Si as substrate and $H_{2}O_{2}$ (**a**) Metal catalyst deposits on Si substrate where $H_{2}O_{2}$ gets decomposed (**b**) Si consumes positive charge and gets oxidized by HF and forms of silicon fluoride-forming Si nano-pillars [102] (published in MDPI open access).

Micro-fabrication can also be achieved by ionic etching through dry-etch and wet-etch methods. Dry-etching is performed in the presence of plasma, while wet-etching is performed in the presence of KOH/HF and oxidizers. Usually, a master mold with micro-/nano-structures is required for patterning the polymer surface via dry-etching, which increases the cost and time of production, while wet-etching is more effective and cheaper with high yields of high aspect ratio structures. In this context, metal-assisted chemical etching (MacEtch) has proven to be beneficial for micro-fabrication in Si at micro- and nano-scales. MacEtch has the advantages of simplicity, low fabrication costs, and the ability to generate high-aspect ratio nano-structures. The mechanism of MacEtch is based on the etching of a patterned metal substrate immersed in a solution of etchant (such as HF or KOH) and an oxidizer, as illustrated in Figure 8 [102]. This constitutes an electrochemical cell that induces an anodic metal etch, where metal atoms are removed at the interface with a catalyst, and a positive carrier has a maximum concentration. As the process proceeds, the catalyst sinks into the substrate to transfer the micro-nano-pattern from the catalyst to the substrate. This process continues until all of the etchant, and byproducts are used out of the pattern.

MacEtch has been used to electroplate Cu and increase the bond strength in semiconductor applications [103] and to micro-machine semiconductor 4H-SiC combined with plasma reactions to make micro-circles and micro-triangles [104], GaAs wafers to investigate etching pits, precursors, and surface passivation [105] and Cu metal-assisted chemical etching (MACE) produced an anti-reflection texture on poly dimethyl siloxane (PDMS) [106]. Although MacEtch has marginal benefits in micro-machining with finished patterns and high-aspect ratios, it does have some shortcomings, including unequal etch rates, and the metal catalyst pattern has some feature size limitations, and micro-porosities can form on the sidewalls. Thus, this process is not particularly suitable for shaping or polishing micro-features.

### 2.6. Reactive Ion Etching (RIE)

**Figure 9:**
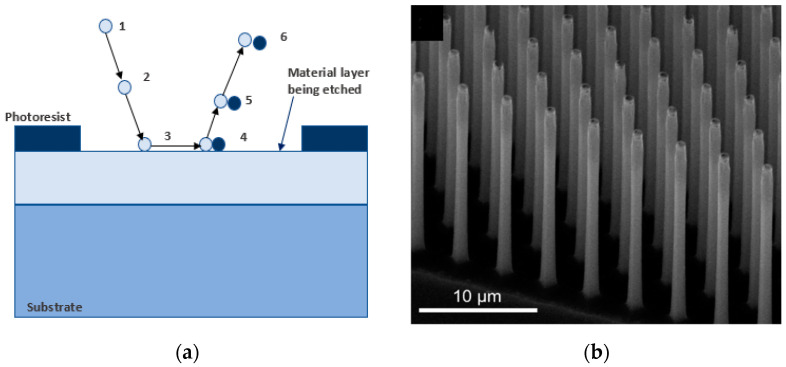
(**a**) Showing the steps of RIE (1: process gases are broken into chemically reactive plasma species, 2: diffusion of reactive species to substrate, 3: absorption of reactive species, 4: reaction between reactive species, 5: desorption of reaction by-products and 6: diffusion of by-products) (after Huff 2022) [113], (**b**) High-aspect ratio Si nano-wires using different mask material during ICP-RIE at cryogenic temperature [110] (open access article distributed under the terms and conditions of the Creative Commons Attribution (CC BY) license).

With growing demand for high levels of anisotropy and high-aspect ratio etching (as shown in Figure 9b) in micro-fabrication, reactive ion etching (RIE) has proven to be a transformative technology. RIE is a combination of both plasma and ion milling with the process pressures in the range of $10^{-1}-10^{-3}$ torr. RIE involves the plasma species imparting kinetic energy into the substrate atoms to initiate etching and chemical reacting species to react with the substrate, which is desorbed from the surface as a by-product. The sequence of operations is shown in Figure 9a. It uses different gases containing halogens (group VII elements) such as ${\mathrm{SF}}_{6},{\;\mathrm{CF}}_{4},{\;C}_{3}F_{8},{\;C}_{2}F_{6,\;}{\;\mathrm{BCl}}_{3}$ and others for etching ${\mathrm{Si}}_{2}O,\;\mathrm{SiC},\;\mathrm{SiN}\;\mathrm{and}\;\mathrm{Si}$, due to their high electronegativity and reactivity. Some of the important considerations of RIE are etch rate (depth of etching per unit time), anisotropy (material etched laterally, which results in undercutting of masking layer), loading effect (etching rate dependent on amount of surface area being etched), and etch lag (etch rate being lower in smaller features than larger ones). There are various forms of RIE, such as inductively coupled plasma reactive ion etching (ICP) RIE, deep reactive ion etching (DRIE), cryogenic DRIE, and Bosch DRIE. These RIE techniques are used to micro-fabricate silicon hollow micro-needles with smooth tapering [107], organic semiconductors with high-resolution displays [108], the optimization of high-aspect-ratio vertical Si nano-wire anodes for lithium-ion batteries [109], and the high fidelity and low roughness of SiC substrates for bulk acoustic wave resonators [110], investigate etching induced damage in p-type GaN at low temperatures [111] and the isotropic silicon etching of MEMS [112]. The process has some drawbacks, however, including being expensive because of the use of complex equipment, controlled conditions, and sidewall defects.

### 2.7. Summary and Limitations of Micro-Fabrication Processes

Micro-fabrication is a state-of-the-art technology for manufacturing miniaturized components such as micro-electrodes, sensors, optics, micro-fluidics, and medical implants, which demand high accuracy and surface finish. Over the years, it has been enhanced through the development of advanced micro-tools, micro-electric discharge machining, laser polishing/ablation, LIGA, electrochemical etching, and reactive ion etching, the capabilities of which are listed in Table 1. However, as feature sizes become smaller by 100 s to 10 s of μm, the ability to control surface finish and geometry (radius) becomes increasingly difficult. The micro-fabrication processes discussed so far are capable of fabricating small features but the tool/workpiece interface (micro-machining), cracks and porosity (electric discharge machining), internal stresses and high cost (laser polishing/ablation), size limitation and unequal etch rates (MacEtch), and rectilinear nature of light (UV/Xray in LIGA) make it difficult to machine around corners and edges. Furthermore, to achieve mirror level finishing requires non-contact finishing technology with well controlled processes. On the other hand, electrochemical polishing is a non-contact micro-finishing process that removes material by macro- and micro-levelling. Principally, it has higher removal rates around asperities than the base regions. Thus, there is clear potential in this process for the shaping and polishing of micro-features with high-aspect ratios and complex geometries.

## 3. Theories of Electropolishing

**Figure 10:**
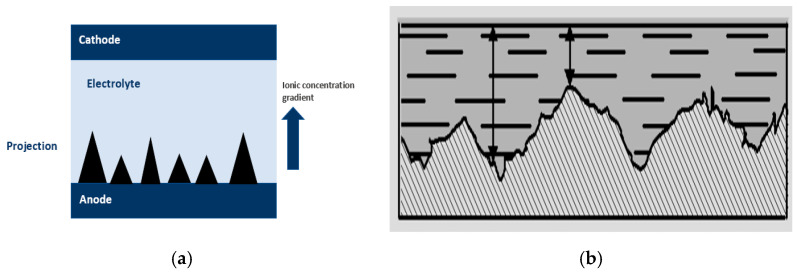
(**a**) The ionic gradient formed when anode (workpiece) and cathode are immersed in electrolyte [70] (after Yang 2017 [70]), (**b**) The variations in current intensity at the peak ($I_{P})$ and recess ($I_{r})$ areas of anode.

Theories of electropolishing provide deep insight into the electrochemistry of the process. It involves the process of ion formation, double layering, and the migration of ions in the electrolyte, when an anode (workpiece) and cathode (counter metal) are subjected to DC/AC supply (as shown previously in Figure 2). The metal surface (anode) is uneven at the start, and there is a difference in the ionic concentration gradient, where the intensity is higher at the peaks than at the recessed areas, as indicated in Figure 10.

Anodic dissolution starts due to the voltage gradient when a voltage is applied to the I–V plateau region. There are different theories that describe this process in terms of viscous or salt film generation (Jacquet), the diffusion of ions (Elmore), acceptor ions (Edwards), and adsorbate mechanisms (Hoar).

### 3.1. Jacquet Theory (Resistance)

The first study of the anodic dissolution of metals was done by Jacquet, who observed that the surface of copper becomes shiny at a specific voltage (V) and current (I) values in a solution of phosphoric acid. This bright and shiny surface of copper is due to the surface leveling of an initially rough surface and the evolution of oxygen. The extent of leveling of the copper depends on the electrolyte concentration, temperature, and current–voltage values. However, after electropolishing, a very thin film of copper phosphate is observed on the surface of the anode [5].

**Figure 11:**
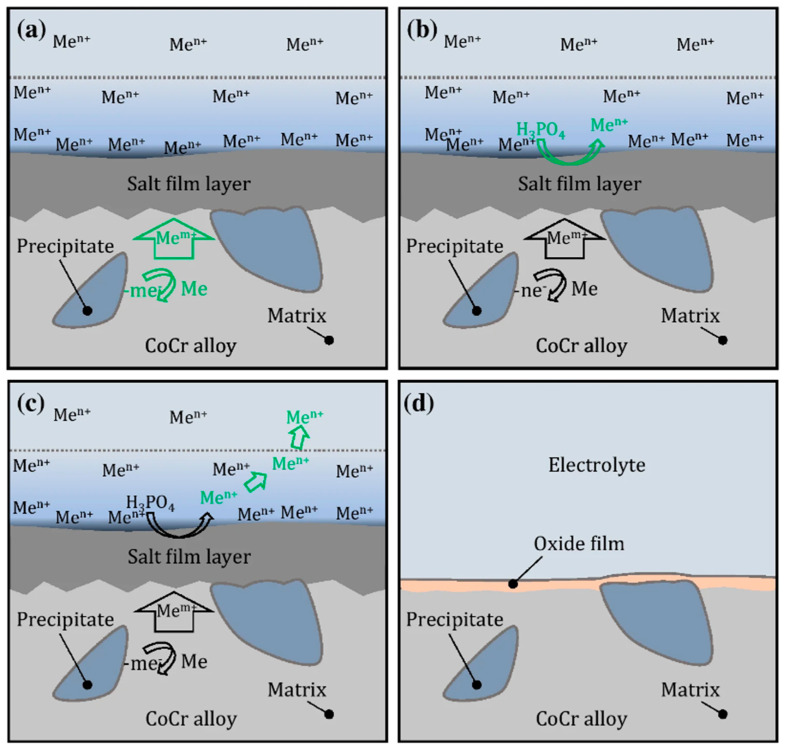
The electropolishing of CoCr dental implants in phosphoric acid, (**a**) initial formation of salt film layer, (**b**) formation of the anode and electrolyte ions, (**c**) movement of anode ions through the salt film, (**d**) an electropolished (levelled) surface of metal with oxide film formed [116].

At the start of electropolishing, a viscous layer forms near the anode and bulk electrolyte with regions of peaks and crevices (due to the surface roughness of the anode, Figure 10). Moreover, the layer has a higher viscosity and electrical resistivity, which serves as a resistance barrier [3,4]. When power is supplied, ions formed at two electrodes migrate through the resistive barrier and form complexes. The process continues until the anode surface becomes smooth due to the variation in current density between the peak and recessed areas. An oxide layer is formed at the end (steps are shown in Figure 11). In this manner, a rough surface becomes level and even.

After copper, other metals were also electropolished, including nickel, aluminum, iron, zinc, lead, and tin in perchloric and glacial acetic acid.

Wei and Fang electropolished CoCr dental implants in phosphoric acid [116]. They observed the formation of salt film at the start when the dental implant surface was uneven, then ions migrated through the film into the solution, until the surface became even, and an oxide layer was formed at the end of the EP process, as summarized in Figure 11.

The effects of electrical resistance, anodic dissolution, and oxide film formation have been studied extensively, e.g., the dissolution behavior of nickel in sulfuric acid (H_2_SO_4_), the oxide layer and corrosion resistance of magnesium in sulfuric acid, hydrogen chloride (HCl), sodium chloride (NaCl), potassium chloride (KCl), sodium hydroxide (NaOH), potassium hydroxide (KOH), and water, and the dissolution of Inconel in potassium citrate (C_6_H_5_K_3_O_7_).

### 3.2. Elmore Theory (Diffusion)

**Figure 12:**
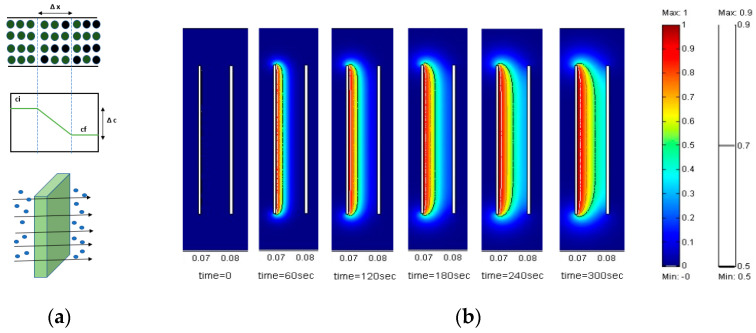
(**a**) The movement of ions from surface of anode based on the concentration gradient developed in electrolytic solution. (**b**) Formation of uniform and thick viscous film at anode for various time intervals at constant diffusion coefficient (D = $10^{-7}\mathrm{cm}/s)$ [118], open access provided by WIT press 2005.

In 1940, Elmore presented the idea of diffusion instead of the migration of ions for the anodic polishing of metals [117]. He explained that polishing occurs when a sufficiently concentrated layer has been established at the surface of a metal. There were two main assumptions associated with his diffusion theory. The first assumption was that, as current passes through the electrolytic cell, a concentration gradient that is formed at the anode and metal ions will leave the anode by diffusion and not migration,

(4) $$i=-AFD\;{\left(\frac{\partial c}{\partial x}\right)}_{x=0}\;$$

where *A* is the surface area of the anode, *F* is Faraday’s constant, *c* is the concentration of metals, and *D* is the coefficient of diffusion of the dissolved metal. The second assumption was that, as the current i passes, the concentration reaches $c_{m},$ which is the maximum solubility limit of metal from time $t_{o}$ to a maximum value at time $t_{m}$. The time progresses from $t_{o}$ to $t_{m}$, and the concentration gradient exists at the anode, as per Equation (4). Thus, the electropolishing of a Cu-$H_{3}{\mathrm{PO}}_{4}$ cell is dependent on the formation of the concentration gradient, where high protruding areas will diffuse more rapidly than recessed ones. This movement of ions based on diffusion can also be described by Fick’s first law [44], Equation (5):

(5) $$J_{i}=-D_{i}\;\frac{\partial c_{i}}{\partial x}$$

where $J_{i}$: flux of species, $D_{i}$: diffusion co-efficient, $c_{i}$: concentration in direction of species, and $\frac{\partial c}{\partial x}:$ concentration gradient (Figure 12a shows the concentration gradient and movement of ions due to diffusion).

Lee and Lai [118] simulated the viscous layer formation and established a numerical model to predict its thickness and uniformity. They found that current distribution, diffusion, and fluid field determine the viscous layer development and surface quality of polished metal. Three coefficients of diffusions D = $10^{-7}\mathrm{cm}/s,\;10^{-8}\mathrm{cm}/s\;,\;10^{-9}\mathrm{cm}/s$ were selected at three speeds, 1 cm/s, 0.5 cm/s and 0.1 cm/s, to keep the same inlet parameters in their numerical model. The formation of uniform and thick layers at different intervals of time is shown at D = $10^{-7}\;\mathrm{cm}/s$ in Figure 12b. Optimum results were predicted to be obtained for a diffusion coefficient in the range of $10^{-7}\mathrm{to}\;10^{-8}\mathrm{cm}/s$ in the presence of a suitable electrolyte.

### 3.3. Edwards Theory (Acceptor)

Edwards in 1953 [119] contradicted Elmore when examining the effect of bulk Cu concentration in a Cu-$H_{3}{\mathrm{PO}}_{4}$ cell. He proposed the acceptor theory, which states that the diffusion of anionic acceptors from an electrolyte (which are ${\left({\mathrm{PO}}_{4}\right)}^{3-},\;{\left({\mathrm{HPO}}_{4}\right)}^{2-},\;{\left(H_{2}{\mathrm{PO}}_{4}\right)}^{-}$ in the case of phosphoric acid). Thus, the diffusion process is based on anionic acceptors into the Cu surface, which is higher at the peaks than at the recessed areas. It is not the diffusion that controls the dissolution of metals; rather, it is the distribution of acceptor anions. This distribution is dependent on current density and electrolyte concentration. He observed that, as the viscosity of the electrolyte is changed, there is no significance to the ionic distribution in the dissolution process.

**Figure 13:**
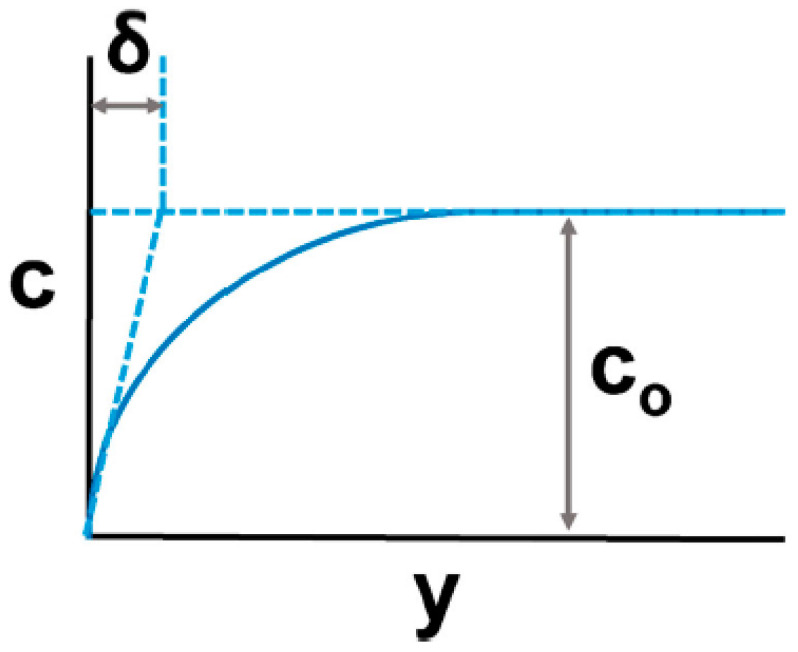
Graph showing acceptor concentration as a function of distance from the electrode, (after Wagner [120]).

Later, Wagner [120] used the acceptor theory to mathematically analyze an ideal electropolishing process. While electropolishing, silver and cyanide ions are acceptor anions forming Ag (CN), and this was represented by a sine wave profile,

(6) $$y=b\sin\left(\frac{2\pi x}{a}\right)$$

where *y*: distance from average surface plane of anode, *b*: amplitude and *a*: wavelength of anode. This growing acceptor concentration is shown in Figure 13.

Roberto, in 1995 [121], used the term “water acceptor mechanism” to describe the behavior of Cu in phosphoric acid. He related the water content present in phosphoric acid to the acceptor ions. In 2004, Du [15] also used the acceptor model to electropolish a Cu/Ta/Si wafer rotating disk electrode in a phosphoric acid solution, and measured a diffusion coefficient that agreed with hydrodynamic impedance. The water acceptor mechanism has been widely accepted and validated to date [122].

### 3.4. Hoar Theory (Passivation)

The limit to which any metal will dissolve in an electrolytic solution was explained by Hoar and others [123,124]. A thin oxidation film forms at the surface of an anode when the surface is electropolished. This film impedes the formation of further metallic ions, even if the supplied potential is increased. This marks the passivation stage in electrochemical polishing. Hence, electropolished metals offer more resistance to corrosion, increasing their importance in industrial applications.

This formed passive layer varies from metal to metal, and depends on the oxidation state of the metal. It is usually very thin, typically in the orders of a few dozen Angstroms. Hoar and others used this concept to explain the non-wettability of mercury as evidence for the formation of an oxide film on copper during electropolishing in phosphoric acid [4]. Some metals are more easily oxidized (Al and Zn) than others (Hg, Cu, Ag, and Pt). The process is more favored by oxidizing agents, while it is diminished by halides or reducing agents.

The anodization of steel, nickel, vanadium, titanium, and aluminum alloys under varying conditions of pH, halides, sulfides, and temperature has been studied to observe the passivation phenomenon [11,123,125]. It was observed that more aggressive ions such as chlorides, halides and fluorides weaken and break down the passivation film. This can cause pitting and affect the corrosion properties of metals in various biomedical products such as stents, and orthopedic and dental implants.

### 3.5. Darmois Theory (Adsorption)

The electropolishing of noble metals such as gold, platinum and palladium was studied by Rowland in solutions of molten salts (potassium chloride and sodium chloride) at high temperatures [126]. It was found that polishing occurred due to the adsorption of ions at the anode, rather than the formation of an oxide layer. As these metals are thermodynamically more stable than baser metals, electropolishing could not be explained by an anodic layer of oxides or hydroxides. Rather, the concept of adsorption was coined, which clarified the non-adhesion of mercury droplets to the copper studied by Hoar and Farthing [4].

**Figure 14:**
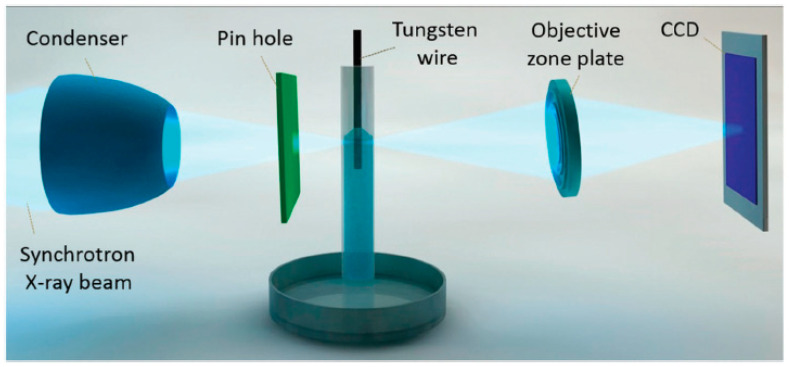
Examination of electropolished tungsten in KOH solution where wire is surrounded by gel-like structure [130]; copyright granted by author (Konstantin, 2015 [130]).

The electropolishing of gold, platinum, and palladium was also conducted at fairly high temperatures—950 °C, 970 °C, and 1020 °C [126]—where the existence of an oxide layer is not possible. Darmois and others presented a theory based on the adsorption of anions on the anode from the electrolyte. In this case, a thin layer of ions forms on the anode surface by means of adsorption, which will dissolve metal; the formation of such a thin layer of ${\mathrm{ClO}}_{4-}$ ions was observed in aceto-perchloric baths [127,128]. The same happens in the case of silver electropolishing in a cyanide solution where no oxide layer is formed [129]. The electropolishing of tungsten in an aqueous solution of potassium hydroxide leads to the formation of a gel-like structure around the anode, based on the concept of ionic adsorption [130], which can be seen in Figure 14.

## 4. Important Factors

In EP, metal is removed from the surface via anodic dissolution, generally by less than 25 μm (0.001 inch), but potentially by more on sharp edges [131]. In order to have an optimum level of macro- and micro-polishing, the rate of metallic dissolution can be enhanced. This depends on some key factors, including electrolyte composition, time, temperature, electrode gaps, and forced convection, which are discussed below.

### 4.1. Electrolyte Composition

The composition of an electrolyte plays a key role in electropolishing. It acts as a conductive and thermal medium for the movement of ions. As the electrodes are immersed in the electrolyte, there is the formation of a double layer (one solid layer at the electrode end and one liquid layer at the electrolyte end), a viscous or resistive film and initial potential will occur, which allows the transfer of ions and metallic dissolution into the electrolyte. The nature of the electrolyte that is used determines the material removal and the surface leveling of the workpiece. Usually, electrolytes are either acidic, basic or ionic liquids, depending on their composition. Furthermore, they may contain organic and inorganic additives to improve their electrical conductivity. Electropolishing involves both macro- and micro-scale smoothing; macro-leveling depends on the electrolyte composition, while micro-leveling depends on the additives.

The origin of electropolishing was marked by the use of sulfuric and phosphoric acid solutions (acid content: 50–80%, surfactants: water/glycerol 30–10%) to reduce the surface roughness (Sa and Rz) of metals and alloys like copper, nickel, steel, titanium, niobium, magnesium, and aluminum [2,14,20,21,22,116,132,133,134,135,136,137,138,139,140,141,142,143,144,145,146,147]. The reason for their widespread use is that sulfuric acid provides a sufficiently large bath conductivity, and phosphoric acid is primarily responsible for the polishing of the metal surface [148]. Some of the commonly used organic and inorganic additives include ethanol, isopropanol, tri ethanolamine, oxalic acid, ethylene diamine, methane sulphonic acid, boric acid, magnesium chloride, and nitric acid.

Increasingly, however, there is a growing trend to use green electrolytes, which are ionic liquids that keep the environment clean and safe [30,31,32,34,36,37,38,39,41,46,147,148,149,150]. Those most commonly used are ethylene glycol, choline chloride, and salt mixtures. Strong acids are capable of destroying industrial equipment, micro-nano-channels, and micro-scale features of products. Furthermore, due to the aggressive nature of such chemicals, they require a high temperature for electropolishing, and may not help in achieving low surface roughness values. In order to ensure human and environmental safety, many researchers now prefer to use ionic liquids instead of strong acids.

### 4.2. Temperature

**Figure 15:**
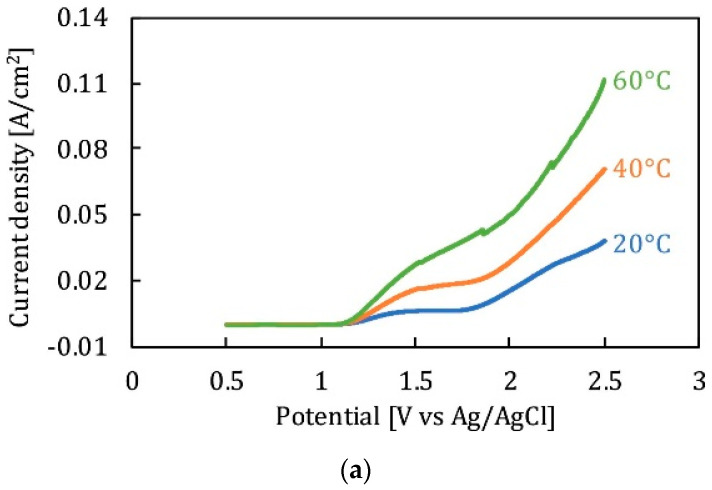

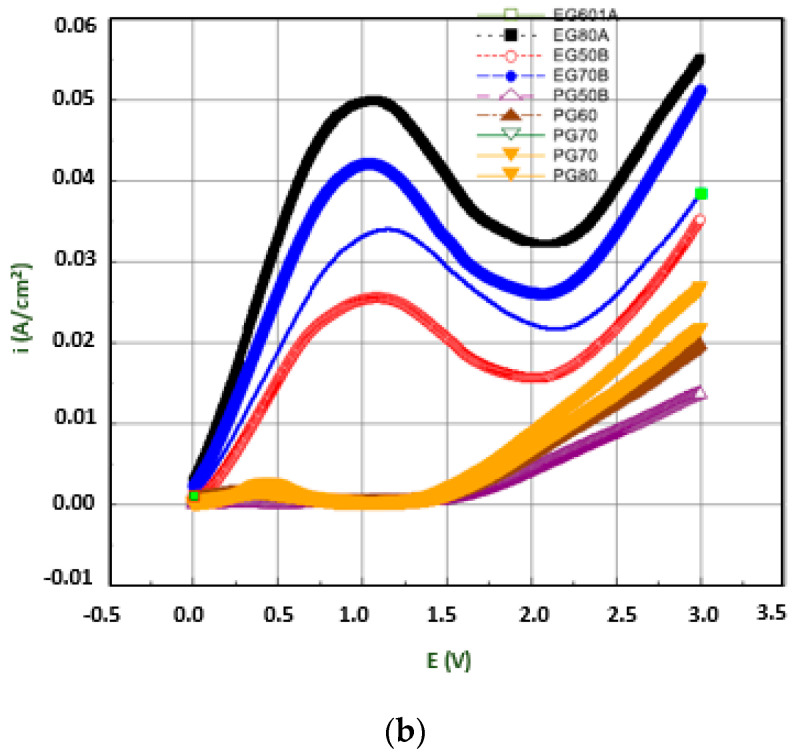
(**a**) The effect of temperature on polarization potential of stainless steel [147], (**b**) Polarization curve for EP of nickel in phosphoric acid and glycerol [151].

Temperature is a significant factor ensuring the mass diffusion and flow rate of ions during electropolishing. As temperature increases, the dissolution rate and current density vary. The effect of temperature is also related to the composition and viscosity of electrolytes. It has been observed that the polarization curve for a given electrolyte increases with an increase in temperature, due to the higher current density, as shown in Figure 15a, where 316 L stainless steel is electropolished in a sulfur free acid solution. Moreover, the formation of a viscous or salt film is dependent on the electrolyte composition which facilitates ionic migration and dissolution. More viscous solutions exhibit lower current densities and material removal rates, while less viscous solutions have higher current densities and material removal rates. This can be seen in Figure 15b, where the electropolishing of Ni in phosphoric acid-glycerol and ethylene glycol-choline chloride has been carried out. Temperature is linked to controlling the aggression of the electrolyte; for strong acids, a higher temperature is required to get surface levelling and shine than compared to those of alkaline electrolytes. This difference was highlighted by Brusov [14], who electropolished aluminum to achieve high optical and mechanical surfaces. He used several electrolytes, some acidic ($H_{2}{\mathrm{PO}}_{4},{\;H}_{2}{\mathrm{SO}}_{4},\;{\mathrm{HNO}}_{3},\;{\mathrm{CH}}_{3}\mathrm{COOH}$), and some alkaline (Na_3_PO_4_, Na_2_CO_3_ and $H_{2}O)$, at varying temperatures (60–115 °C). The electropolishing results were better for an acidic composition ($H_{3}{\mathrm{PO}}_{4},{\;\mathrm{CH}}_{3}\mathrm{COOH},{\;\mathrm{HNO}}_{3}$) at 100 °C with a current density of 30 A/d$m^{2}$.

### 4.3. Wave Form

**Figure 16:**
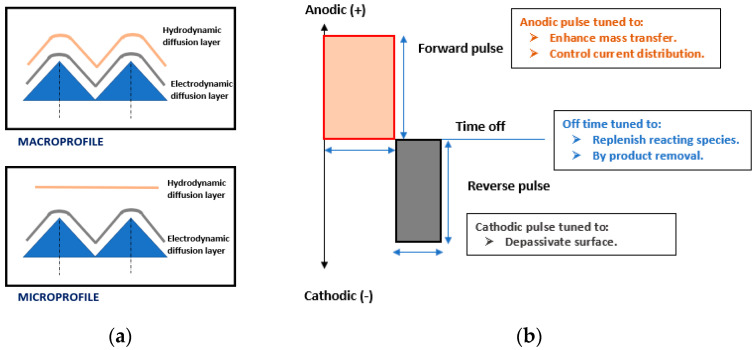
(**a**). Hydrodynamic layer of micro- and macro-profile, (**b**) Generalized pulse/pulse reverse waveform for electropolishing (after Taylor 2001) [53].

The electrochemistry of polishing involves kinetics and mass transportation phenomena. A given wave form provides the cathodic and anodic pulses, which facilitates the formation and dissolution of metal ions. Initially, a hydrodynamic diffusion layer is formed on the anode (due to roughness and non-uniformity) within the electrolytic solution, as shown in Figure 16a. As the current is supplied, there is an electrodynamic diffusion layer which ensures both macro- and micro-level polishing. Conventionally, a DC waveform is applied to metals with flat surfaces, while the electropolishing of strongly passivating metals (niobium, titanium and their alloys) [152] and features/forms [57,153] requires pulse/pulse reverse (PPR) and pulse currents (PC). The PPR has two pulses, one forward and the other off, while PC has forward pulses that are repeated after some time. Both PPR and PC waveforms are used to control material removal rate and current density. Usually, the forward pulse enhances mass transfer and current distribution, while the off pulse (cathodic) helps in replenishing material species, and removes the byproducts, as shown in Figure 16b [53].

Different waveforms can be used to achieve different electropolishing results. More particularly, they can be helpful to electropolish micro-nano-features as the reverse pulse would assure de-passivation, while the off pulse would help to remove by products and to replenish species. It can be seen in the electropolishing of complex SLM features [57] that changes in waveform influence the material removal rate and enhance the control of inner features with higher current densities.

### 4.4. Electropolish Time

**Figure 17:**
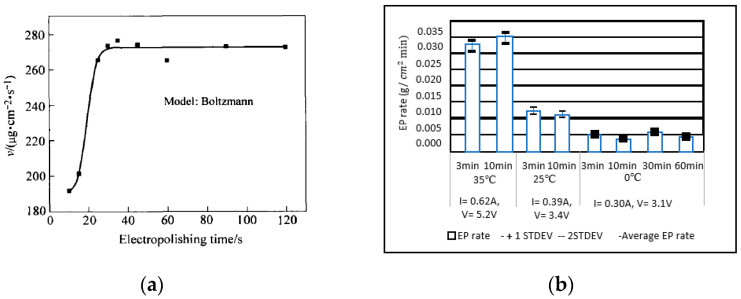
(**a**) The effect of electropolishing time obeys sigmoidal law [154], copyrights granted by Nonferrous Metals Society of China 2006, (**b**) EP rate of Co-Cr samples in phosphoric acid at temperatures of 35 °C, 25 °C, and 0 °C (after Aihara 2009) [140].

EP time is related directly to the amount of material removed; this can be seen from Faraday’s equation. In the presence of optimum electrical conductivity and voltage range, an increase in time will ensure more metallic dissolution and a higher polishing rate. Generally, it follows a sigmoidal relationship [154], as shown in Figure 17a, which shows that the material removal rate increases up to a particular point when other conditions are favorable. Aihara [140] investigated the effect of temperature and time for electropolishing Co-Cr alloys for surface characterization and biocompatibility. The bath temperature was decreased from 35 °C to 0 °C, and polishing time was increased from 3–60 min (Figure 17b). Smooth and hydrophilic surfaces were obtained by increasing time. Controlled EP effects found that lower temperatures and more time resulted in biocompatible and non-toxic Cr-Co samples.

**Figure 18:**
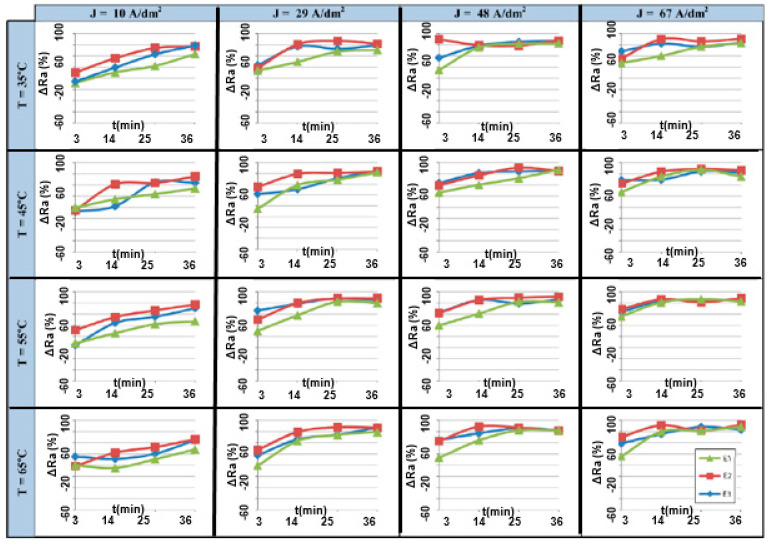
Influence of electrolyte, temperature, and current density of electropolishing of SS [20]; copyright granted by Elsevier.

The significance of current density and time was studied by Núñez, who electropolished stainless steel in varying proportions of phosphoric, sulfuric and chromic acids. Increasing treatment time to 25 min and current density to 48 A/dm^2^ significantly improved surface finish (Ra), reaching maximum polishing ranges of 80–90% [20], as shown in Figure 18.

### 4.5. Electrode Gap

**Figure 19:**
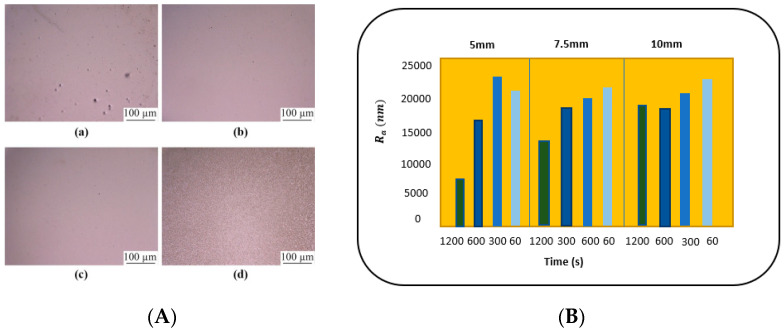
(**A**) Electropolishing of W in environmentally friendly NaOH with electrode gaps of (**a**) 0.15 mm, (**b**) 0.5 mm, (**c**) 1.0 mm, (**d**) 1.5 mm; (**B**) Time and distance Vs roughness (Ra) plot comparing the impact of differing polish times and inter anode/cathode distances at constant temperature and voltage for additively manufactured Ti (after Lassell 2016) [62].

The size of the electrode gap is significant for maintaining current density and surface finish and for avoiding bubbles on the work metal. Usually, current density is increased by reducing the gap between the anode and cathode. This ensures the maximum dissolution of metal within the electrolyte and reduced surface roughness. However, too small a gap would lead to the formation of pits and bubble marks on the metal surface. Han and Fang [46] electropolished tungsten rods (1 mm dia) in NaOH, for various electrode gaps of 1.5, 1, 0.5, 0.15 mm. The results can be seen in Figure 19A: as the distance between electrodes is reduced, the surface finish improves, but too small a distance leads to pits forming on the surface. Optimized results were obtained at a 0.5 mm inter-electrode gap: current density was suitable and bubbles were also avoided on the surface of tungsten. Lassell checked the effect of the inter-electrode gap on EP additively manufactured ${\mathrm{Ti}}_{6}{\mathrm{Al}}_{4}V$ titanium targets [62]. The purpose of this work was to improve fatigue life and surface quality for Ti alloy applications in engine and structural components. The design of experiments based on the variation of electrode gap and electropolishing time is shown in Figure 19B. Two conclusions were drawn: the decrease in electrode gap (from 10 to 5 mm) increases current density and reduces the surface roughness, while an increase in time (60 to 1200 s) helps in removing surface unevenness. This enhanced the fatigue strength of Ti_6_Al_4_V.

### 4.6. Hydrodynamic Conditions (Forced Convection)

**Figure 20:**
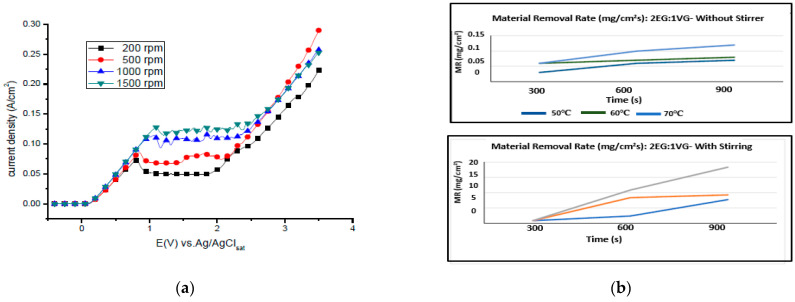

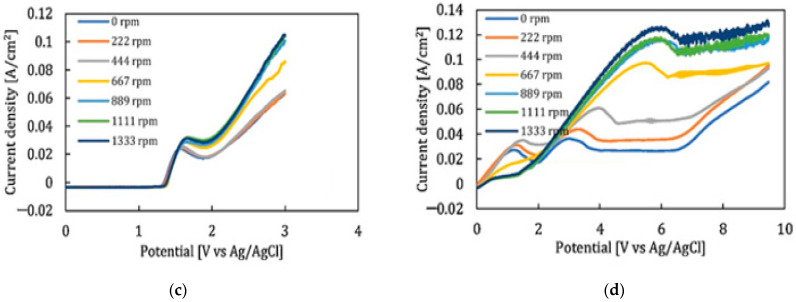
(**a**) The increase in current density with magnetic stirring while electropolishing brass in 70% phosphoric acid [21], (open access article distributed under the terms and conditions of the Creative Commons Attribution (CC BY) license). (**b**) A comparison of material removal rate with and without magnetic stirring in electropolishing of nickel mold inserts in phosphoric acid and ethylene glycol [151], (**c**) Polarization curve for 316SS with magnetic stirring for acidic (H_2_SO_4_-based) and (**d**) green (NaCl-based electrolytes [30]). Note: the different colour lines in (**a**,**c**,**d**) correspond to different stirring speeds, while in (**b**) they correspond to different temperatures.

All four stages of the electropolishing process, namely, etching, passivation, electropolishing and gas evolution, are linked to the flux of ions, temperature, electric conductance, and appropriate electrode gap. To promote electropolishing, forced agitation can be important. This reduces the chances of pitting and excessive gas formation at the anode end. This can be seen in work by Ching [21], who electropolished brass in 70% phosphoric acid with (rotating cylinder electrode) to observe the formation of a ${\mathrm{Cu}}^{+2}$ rich layer on brass. The convection force was increased by increasing the rotational speed from 200 to 1500 rpm. This served to increase current density and improve surface quality (as shown in Figure 20a). However, at speeds below 500 and at 1500 rpm, pit formation was observed. Similarly, a higher MRR (material removal rate) was investigated by Chandralal [151] for the electropolishing of a nickel mold with and without agitation. The results highlighted in Figure 20b show that the material removal rate improved significantly with the help of magnetic agitation. Although electropolishing occurs in static conditions, convection helps to ensure the proper diffusivity and transportation of these ions, and accelerates the removal of burrs and particles from the surface of a workpiece (as shown in Figure 20c).

## 5. Micro-Nano-Scale Polishing—Issues and Trends

The electropolishing of micro-nano-scale features is more challenging, as it involves controlled material removal rates, precipitation, and smooth and mirror-like finishes on various forms and channels. Care is required to maintain the channel integrity and design. There has been much use of micro-nano-scale metal electropolishing, e.g., radiofrequency electromechanical systems (RF MEMS), additively manufactured parts (based on SLM, EBM, and LPBF, etc.), sample preparation for transmission electron microscopy (TEM), superconducting niobium radio frequency (RF) parts, bio-medical stents, and transplants. As these parts involve small asperities with features that have dimensions less than 1 um, a high-viscosity electrolyte is preferable and material removal is faster at peak regions than from recessed areas. The micro-nano-pattern formation depends on the orientation, and the presence of overhangs or a lattice structure in the part being electropolished.

### 5.1. Metal AM Parts

**Figure 21:**
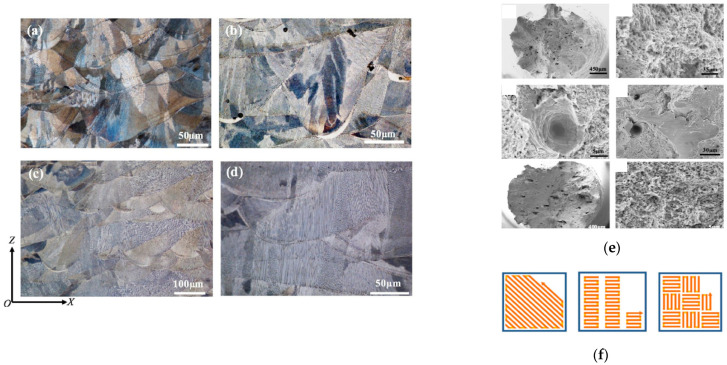
(**a**–**d**) High magnification of micro-graphs showing micro-structure of 316 L samples fabricated at 200 W using different scanning strategies, (**e**) Additional SEM micro-graphs, (**f**) Schematic of different laser scanning strategies, i.e., Meander, Stripes and chessboard [61] (open access article distributed under the terms and conditions of the Creative Commons Attribution (CC BY) license).

Additively manufactured parts offer design freedom, complexity, and cost benefits for micro-scale features. The machining of complex geometries and hard materials is challenging for high-performance materials such as steel, titanium, tungsten, nickel, and their alloys, which is why AM is attractive. However, the surface roughness, internal porosity, grain structure, and fatigue strength of AM parts can be the cause of problems, for which electropolishing offers a good solution. Electropolishing has increased the industrial applications of metallic AM parts in the aerospace, automotive, and medical device industries, due to the improvement that it provides for surface quality, controlled material removal, and mechanical strength [46,155,156,157]. There are two basic metal additive manufacturing techniques, namely powder bed fusion (PBF) and direct laser deposition (DLD), which involves a heat source to melt or sinter the processed material with laser power, speed, and cooling rate determining the quality of the manufactured part. Qiu and Al Kindi [61] electropolished SS 316 samples using selective laser melting (SLM) to find the dislocation and twinning within nano-needles. The distribution of pores was studied with different laser scanning strategies (chessboard, meander and strip) with island sizes of 1 × 1 mm and 5 × 5 mm, as shown in Figure 21. The results suggested that laser power is more influential than scanning strategy during SLM. 

The additively manufactured 316 L parts had superior strength and ductility compared with conventionally manufactured counterparts. Samples of 3 mm diameter and thickness of 150–200 μm were electropolished in a solution of 5% perchloric acid and 95% ethanol for TEM-EDX at 200 kV. The samples had a high cooling rate after SLM, which led to the formation of Si- and Mn-oxides [61].

**Figure 22:**
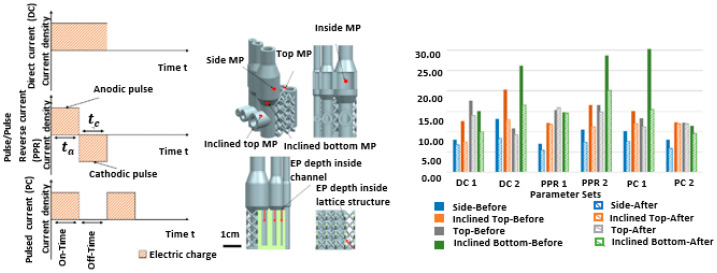
The electropolishing of complex steel SLM parts with various waveforms, geometric details and average mean height Sa [57] (open access article distributed under the terms and conditions of the Creative Commons Attribution (CC BY) license).

**Figure 23:**
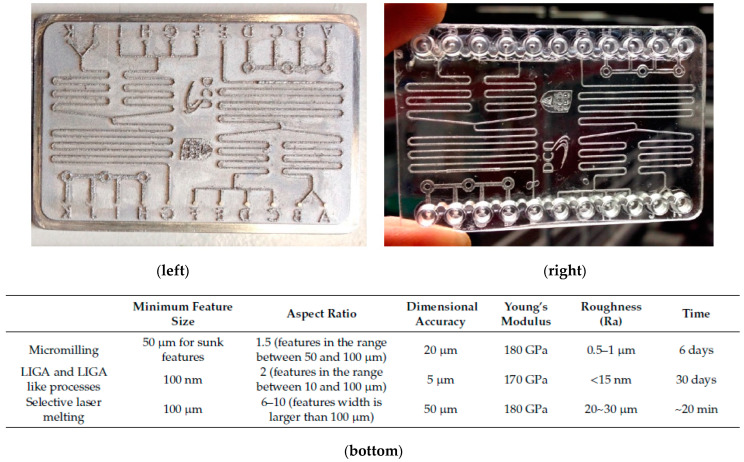
Micro-patterns printed on steel substrate using SLM (**left**) and polymeric micro-fluidic chip after micro-injection molding (**right**) along with a comparison of other micro-fabrication processes with SLM (**bottom**) [60].

In work by Hu et al. [158], graphene-reinforced copper matrix composites were fabricated using a laser adaptive process. A 4140 structural steel plate was used as a substrate with dimensions of 10 mm × 8 mm × 2.5 mm, the copper powder having a diameter <10 μm, and multi-layer COOH-rich graphene nano-platelets with average diameters <2 μm were used. The twin jet electropolishing of Gr-Cu was used, where twin-jet liquid managed to corrode copper, but not graphene. The samples were observed using TEM, which showed the growth orientation for pure copper and Gr-Cu composites as (111). Furthermore, nano-hardness indentation tests of Gr Cu and copper were 135 HV and 110 HV, which represent an increase of 22.7%, and thus graphene has ultra-high strength mechanical properties. Ferchow et al. [57] applied electropolishing (with DC, PPR and PC) to complex structures manufactured by SLM. Their samples were built with a layer thickness 50 m, hatch distance 0.105 mm, and laser power 290 W. The electric current was kept constant at 12 kC/dm^2^, with an average current density of 30 A/dm^2^. Of the various waveforms that had been formed, the direct current gave good material removal and surface roughness values with high average current density ACD, as shown in Figure 22. Zhang et al. [60] manufactured micro-mold patterns using an SLM technique on a pre-finished substrate for micro-plastic injection molding. Thus, a micro-injection mold tool insert was developed for manufacturing cyclic olefin copolymer (COC)-based micro-fluidic chips which were successfully used to monitor the nitrate concentrations in environmental water. SLM was compared with other micro-fabrication processes, namely micro-milling and LIGA, in terms of dimensional accuracy, aspect ratio, Young’s modulus, roughness, and manufacturing time. It was found that SLM is a far faster mold tool development process, although it has limitations of dimensional accuracy (50 μms) and surface roughness (20–30 μms), as shown in Figure 23 [60].

Numerous biomedical implants are additively manufactured in steel, chromium, cobalt, and titanium. The major concern, however, with metallic AM parts is their microstructure, caused by porosity or partially melted powder, which limits their mechanical strength and associated structure and fatigue properties.

Direct metal laser sintering (DMLS) has been used to manufacture high-density Ti alloy implants that can be used, as well as conventionally machined ones, although there can be issues of fatigue strength due to crack generation, insufficient sintering, and entrapped gases. A change in orientation direction and scan line distance can resolve these issues. SLM-manufactured parts can be prone to diffusional problems and the existence of lamellas of α-phase in a β-phase. Such issues may be minimized by controlling the printing parameters and avoiding diffusion around the corners of implants. EBM-manufactured parts have ideal surface structures for biomedical applications in comparison to conventional ones. However, they too can have issues of porosity and elongation of the micro-structure grain, although shot peening can improve the reduced mechanical strength of such parts [12].

In recent work with an Inconel 718 super alloy, which was manufactured using the PBF technique, specimens were electropolished with an exposed area of 1 ${\mathrm{cm}}^{2}$. In order to avoid passivation oxide film, a pulse current was used to achieve a higher anodic pulsed current density. Electropolishing was performed using HClO_4_–CH_3_COOH with variable current densities, and temperature which resulted in reduced surface roughness compared to both printed parts and heat-treated parts [159]. Hence, electropolishing proves to be a good finishing technique for polishing AM parts by decreasing porosity and surface roughness, and thereby increasing fatigue life and corrosion resistance.

### 5.2. Biomedical Stents and Implants

**Figure 24:**
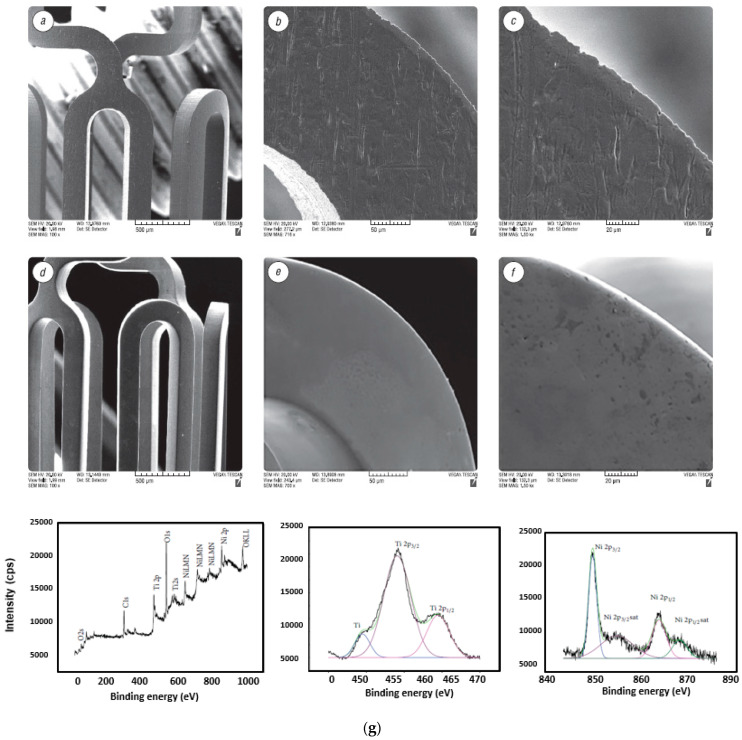
Micro-photographs of stents before (**a**–**c**) and after (**d**–**f**) EP showing scratches, oxide and slag [56]; (**g**) XPS analysis of mechanically polished Nitinol implant [162] (open access article distributed under the Creative Commons Attribution License).

The complex geometry, small size, and low rigidity make the finishing of stents and implants difficult to achieve with mechanical micro-machining. Usually manufactured through a laser cutting process or μ-EDM, all sharp edges, heat affected zones and burrs need to be removed (as shown in Figure 24a–f). Electropolishing has proven to be useful for controlled material removal and improving surface finish in dental pulp needles, orthopedic implants, and bio-medical stents. Optimized parameters of EP, current density, polishing time, current pulse (DC, PC and PPC), and electrode gap lead to good surface quality, thus making them corrosion-resistant, smooth, and hydrophilic, connective for tissue attachment, and more biocompatible [7,17,54,160,161]. Still, there are some issues of lower corrosion stability and hazardous elements in the formed oxide layers which need to be addressed.

Nazneen et al. [6] examined medical grade 316L stainless steel under different electropolishing conditions: 4.5 M $H_{2}{\mathrm{SO}}_{4}$ + 11 M $H_{3}{\mathrm{PO}}_{4}$ proved best in surface nano-texturing. Best results were obtained with a potential of 2.1 V for an EP time of 10 min at 80 °C. Surface topography was also measured to check roughness values, the lowest of which was found to be 0.4 nm, while AFM and XPS analyses revealed the presence of Cr, P, S, O, Mo, and Ni after the EP process.

Bhuyan and Brandon [54] worked on the polishing of cardiac stents with thicknesses of 50 and 100 microns, and initial surface roughnesses (Ra) of 220 and 110 nm. These stents are typically implanted in arteries and need to be smooth and burr-free, and have optimum flexibility to avoid thrombosis and neointimal hyperplasia. Samples of austenitic stainless steel 304 and 316 L were polarized in an electrolytic bath of sulfuric and phosphoric acid with DC and PC (average current density—23 mA/${\mathrm{cm}}^{2}$). Improvements were sought in both surface roughness, amount of pitting, and surface brightness. The resulting stent improved in surface roughness 50 nm and was rich in Ni and Cr content, ensuring good corrosion resistance and mechanical strength. Chembath et al. [155] conducted an in vitro analysis of more corrosion-resistant and biocompatible NiTi alloy by electropolishing in perchloric acid at low temperature. NiTi is usually preferred over stainless steel due to the shape memory effect, flexibility and superior bio-compatibility. The samples prepared were vacuum arc melted and hot rolled strips of 10 mm width and 1 mm thickness that were etched in a solution of HF and HNO_3_ and was later electropolished in perchloric acid at 15, 20 and 25 V. The change in corrosion rate was calculated by impedance spectroscopy (EIS) using a CHI604D electrochemical workstation, and was also given analytically as:

(7) $$\mathrm{Corrosion}\;\mathrm{rate},\;C=\frac{\left(K\;\times i_{corr}\;\times \;EW\right)}{\rho }$$

where *K* = constant, 3.27 × 10^−3^ mm⋅g/μA⋅cm⋅year, *i_corr_* = corrosion current density in μA/cm^2^, *EW* = equivalent weight of NiTi alloy in grams, and *ρ* = density of NiTi in g/cm^3^.

Current trends for electropolishing are now changing from the use of conventional electrolytes which can be environmentally hazardous, toxic, and sometimes explosive, to the use of green ionic liquids. Kityk et al. [36] recently electropolished titanium alloys in deep eutectic solvents (DES) to achieve improved corrosion resistance, osseointegration, and increased service life. The Ti alloy had small amounts of C and Al, (the presence of Al is beneficial due to its heat resistance, creep resistance, elastic modulus and reduction in hydrogen embrittlement). A deep eutectic solvent was prepared with choline chloride and ethylene glycol in a ratio of 1:2 at 70 °C for 1 h, and electropolished at voltages of 3 and 4 V for 30 and 40 min. Surface roughness and wettability tests were performed subsequently. The samples were also examined with AFM, EDX, SEM, and Raman spectroscopy to check hydrophilicity and composition. Surface roughness was seen to reduce to 68 nm, and there was a significant reduction in the water contact angle, which is favorable for smooth implants, to which more tissue can attach easily. So, electropolishing provides improved quality biomedical implants and stents in comparison to laser polishing, μ-EDM, and micro-machining.

### 5.3. Sample Preparation for TEM

Electropolishing is crucial for TEM preparation investigations of the micro-structure of materials and their crystallinity at a micro-nano-level, the stability of passive films, chemical homogeneity, and the composition of various alloys [11,58,61,158]. It is very suitable for preparing thin metallic foils (~100–150 nm) for TEM analysis [59]. TEM has revolutionized technology for scientists and researchers in the field of nano-materials, and enables the characterization of their unique properties. It has allowed for the investigation of micro-structures, crystallographic information, the initiation of pits, passive film, and corrosion at the nano-structures. Usually, a metal alloy is cut into thin slices with a low-speed diamond saw in the presence of fluid, and a counter load is adjusted so that a sample has an appropriate load for cutting. These discs are mechanically lapped/polished to a thickness of 100–300 μm. Then, they are subjected to twin jet electropolishing, whereby the central thinning of a sample is done to make it transparent to electrons (as shown in Figure 24). In 1996, Aebersold et al. [163] prepared TEM samples by twin jet polishing and rotating electrodes of nickel base super alloys of γ-phase separated by the γ’ solution-matrix. The electropolishing was performed in a mixture of perchloric acid and methanol at 3 V and 7 V, T = 10 °C, v = 400 rpm. Both methods of electropolishing could not be compared directly, as one involved a convection rate, while the twin jet method involves a variable potential rate. The applied voltage plays a vital role in the preferential dissolution of the γ’ phase and poses some difficulty in producing high-quality foils for TEM.

**Figure 25:**
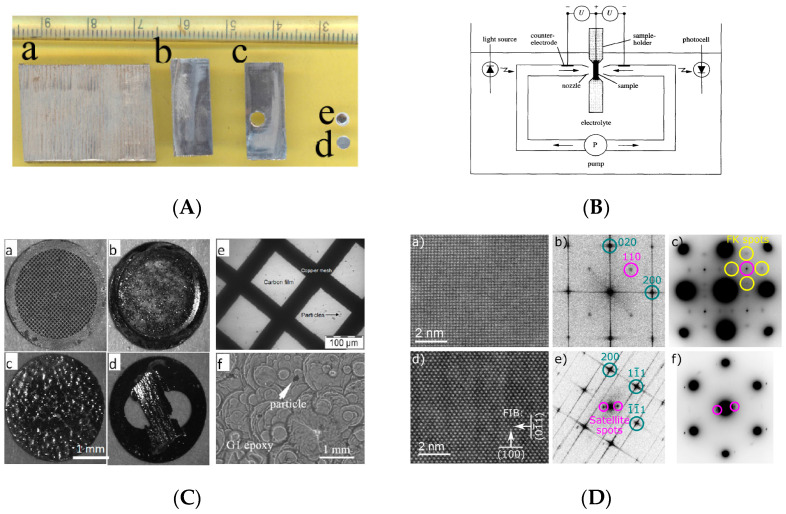
(**A**) Optical images of (**a**) sample, (**b**) slice sectioned from the sample using low-speed diamond saw (isomet), (**c**) slice after punching TEM disc (**d**) TEM disc (**e**) TEM disc after grinding and electropolishing (note the shiny contrast in the central portion due to electropolishing [59] (copyrights granted by author). (**B**) Schematic of twin jet electropolishing, (copyrights granted by Elsevier) [163] (**C**) Nano-structure based optical images of TEM samples in various forms is shown, (**a**) carbon coated, (**b**) mechanically milled, (**c**) free standing ribbon processed by rapid solidification, (**d**) small ribbon bonded to a slotted copper grid, (**e**) TEM image showing the powder particles on copper grid, (**f**) optical image of iron powder particles embedded in epoxy [59] (copyrights granted by author), (**D**) TEM results showing grain orientation of electropolished Al samples at 200 kV electrons, (**a**,**d**) cut-outs from larger ADF-STEM images in [001] and [011], (**b**,**e**) Fourier transforms, (**c**,**f**) diffraction patterns of (**a**,**d**) [29], (open access article distributed under the terms of the Creative Commons CC-BY license).

Rao et al. [59] conducted a review to investigate the crystallographic structure of various metals and composites at the nano-scale level, as shown in Figure 25. The samples were prepared by focused ion beam (FIB) milling and subjected to twin jet electropolishing, which can grip samples of 3 mm diameter with two nozzles positioned in front of each other, and act as a cathode. Perforation occurs at the center, and is identified by using a light source/sensor with the desired thickness of a TEM specimen for electron transparency. In order to electropolish multi-layer samples, a dimpling process is performed. This is done to adjust the thickness of samples, where a constant speed of 3 rpm is used with a grinding wheel of bronze, diameter 15 mm. A built-in micro-meter and dial indicator help in setting the depth using light microscopes.

As with electropolishing composites, ceramics, and brittle materials, this is difficult due to variable etching rates, functional coating, and electrical conductivities for TEM sample preparation [59].

Recently, Wenner et al. [29] used electropolishing to prepare smooth and transparent TEM samples for studying the copper enrichment of aluminum alloys. The precipitate phases of Cu in Al were revealed by electropolishing discs to a thickness of around 80 um, using an electrolyte with 1/3 nitric acid in methanol at a voltage of 20 V. For mass spectrometry, a 30 kV Ga source was used with a scanning area of 100 μm × 100 μm under static conditions, while depth profiling was done at 3 keV over a 600 μm × 600 μm area for a duration of 10 s. This allowed Cu-rich layers to be observed by TEM in both alloys, the second alloy having more copper content.

### 5.4. Semiconductor and Radio Frequency MEMs

**Figure 26:**
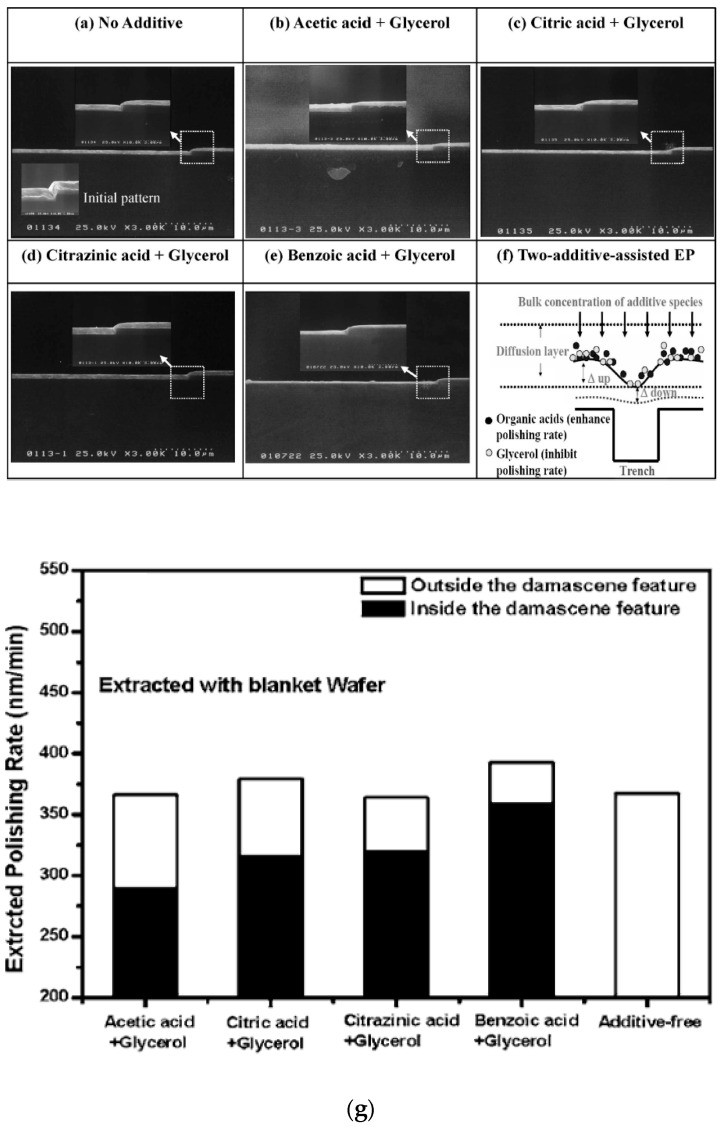

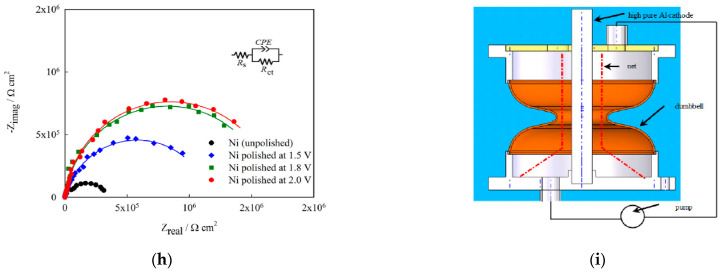
SEM images of damascene for 50 μm patterns with Cu electropolishing at 1.75 V, (**a**) additive free, (**b**–**e**) four two additives (various organic acid + glycerol) electrolytic mixture, (**f**) illustration of two additive EP within damascene feature, (**g**) polishing rate on the inner and outer sides of damascene [166], (**h**) Nyquist plot showing enhanced corrosion of Ni in DES after EP at various voltages [35], (**i**) Schematic for 9 cell super conducting RF Niobium [23] (copyrights granted by Elsevier).

Semiconductors, RF MEMS, and superconducting devices have narrow channels and trenches with high-aspect ratios, internal forms, and very smooth surfaces. They are manufactured through electroforming, mechanical grinding, lapping, and micro-machining, which leads to burr formation, planarization, and internal surface quality issues [13,23,25,33,164,165,166,167]. Electropolishing has the potential to remove burrs and induced stresses, as well as to smoothen surfaces. Kissling [165] electropolished the micro-features of Ni RF MEMS with aspect ratios of up to 40:1. Such devices are sensitive to height variations and have electrically capacitive structural features. Hence, careful material removal is required for channels with widths as small as 10 μm. Ni micro-patterns were polished with a current density of 80 A/dm^2^, with material removal rates of 0.2 to 1.8 μm/s, by using 100 mm silicon wafers metalized with a 3 um titanium layer as a substrate. The planarization of the wafer was achieved by a lapping process, along with electropolishing. Metal removal rates were dependent on the layout and resist, with a chamfering effect at the corner of channels. The corrosion behavior of Ni in deep eutectic solvent was determined by Protsenko et al. [35]. Electrochemical impedance spectroscopy (EIS) was used to check the corrosion resistance. Initially, Ni was electropolished in ethylene glycol and choline chloride (66.67 mol.% and 33.33 mol.%, respectively) for 30 min, at 40–50 °C, at a potential of 1.5 V. The resulting surface had enhanced corrosion resistance in 3% NaCl, and the surface was homogeneous. Liu et al. [166] planarized a patterned Cu wafer in metalized damascene by electropolishing it in a two acid mixture, with the addition of different accelerators in the form of acetic, citric, citrazinic, and benzoic acids, as shown in Figure 26a-g. The wafer consisted of 30 nm-thick ionized metal plasma as a diffusion barrier, a 200 nm-thick Cu film, and 1.7 um-thick electroplated copper as a conductive layer. This was electropolished in a mixture of phosphoric acid and sulfuric acid, along with additives.

Different additives have different polishing rates on Cu-damascene, with the viscous resistance of the passivation layer at the reacting interfaces. The reaction was more effective at the bottom and opening of the damascene. Weak acid at high concentrations enables the performance of accelerators in developing a resistive layer, and decreases local acidity. Hence, acetic acid has a superior performance to other electrolytes [166].

Niobium is used to manufacture super conducting devices with surface finishes that are in the range of 40–50 nm for the RF range. Chu et al. [33] examined the electropolishing of Nb in an HF-based electrolyte 1:2:1 M ratio of choline chloride: urea: ammonium fluoride. A pure Nb wire of 2 mm diameter was used as the working electrode as RDE, an Al plate (10 mm × 10 mm) was used as the counter electrode (CE), and Ag/AgCl as a reference electrode. The grain structure and preferential orientation were also changed and examined before and after treatment. An XPS/EDX analysis revealed that the Ar+ sputtering depth increased, the content of low valence NbO, increased, high-valence ${\mathrm{Nb}}_{2}O_{5}$ decreased, and middle-valence ${\mathrm{NbO}}_{2}$ increased first, and then decreased. The resulting surface was smooth and had a mirror polish finish.

## 6. Electrochemical Shaping of Micro-Structures

### 6.1. Fundamental Principle

**Figure 27:**
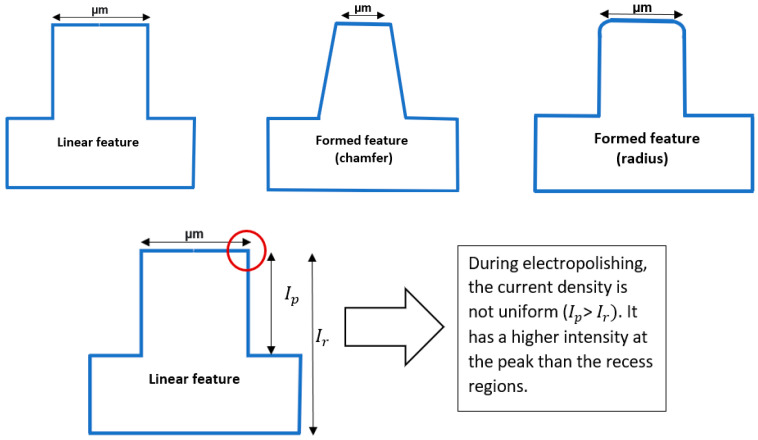
Electropolishing for shaping of micro-scale features.

The working principle of electrochemical polishing has been explained by various theories of resistance [5], diffusion [117], acceptor ions [122,168], passivation [123,124], and adsorption [127]. Initially, when the metal (anode) is immersed in an electrolyte, a double layer is formed (layer of ions—one at the electrode, and the other at the electrolyte side). There is a gradient difference due to the uneven surface of the metal, which causes a higher current density to exist at peak regions than at valley areas. Consequently, more material will be removed from corners and edges when micro-features are electropolished. This uneven material removal rate can cause the shaping of micro-features. Subsequently, the linear micro-feature can acquire a chamfer or radius, as illustrated in Figure 27.

### 6.2. Shaping of Micro-Channels

**Figure 28:**
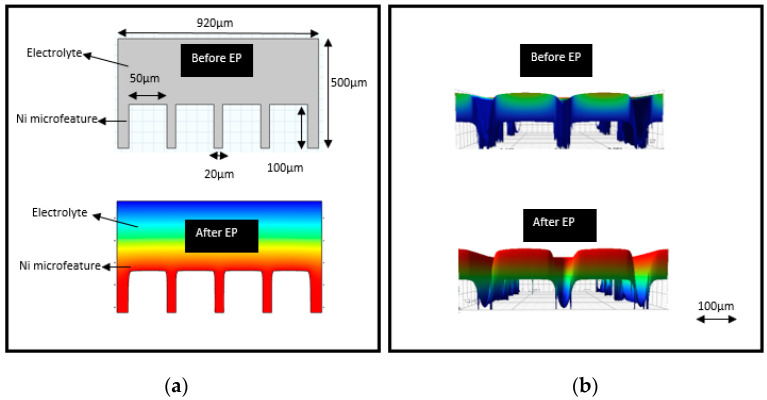
Electropolishing of Ni micro-features (**a**) numerical model simulated in COMSOL, (**b**) experimental results of electropolishing (long channels with 200 μms depth) in a mixture of phosphoric acid and glycerol (work of present authors).

To evaluate the concept of electropolishing for the shaping/forming of micro-features, we have developed a numerical model in COMSOL and undertaken some associated experiments. A 2D numerical model of Ni was created with micro-features. Two physical equations were used to represent the deformed geometry (dg) and electric currents (ec). The 2D geometry of Ni with micro-features is shown in Figure 28a. The complete simulation was computed after assigning appropriate material and boundary conditions, and selecting a suitable time-dependent solver. The electropolishing of Ni was found to result in the shaping of micro-channels, which is due to higher current density at the corner than at the bottom region, as shown in Figure 26a. After the confirmation of our numerical study, we performed electropolishing on nickel samples with micro-channels (long channels 50 × 2100 × 100 μms) in a mixture of phosphoric acid and glycerol. At the working conditions of 2–2.5 V, 50 °C, and a current density of 16.2 A/dm^2^, a clear shaping phenomenon was observed. Figure 28b shows the micro-channels of Ni before and after EP. It is clear that the higher current density at the corner and edges dissolved more metal, and shaped the micro-channel features.

## 7. Conclusions

Electropolishing is widely used in many industries: semiconductor, super alloys, biomedical, radio frequency, and precision manufacturing from the macro- to nano-scale. In this review, the electropolishing and shaping of micro-features have been discussed principally in terms of developing precise micro-scale features. Although micro-machining, laser polish/ablation, μ-EDM, etching and lithography have the ability to manufacture precision micro-parts, they do not offer any equivalent potential for the shaping/forming of a feature. To elucidate electropolishing fundamental theories, important factors and micro-feature applications have also been discussed. The concept begins with the removal of surface defects, burrs, the understanding of oxidative film, and electrochemical impedance spectroscopy (EIS) for corrosion resistance, resulting in smooth and shiny surfaces.

To optimize the process selection of electrolytes, temperature, voltage, current density, electrode gap, and magnetic agitation all play a significant role. Moreover, there is a growing trend to work with greener electrolytes (ionic liquids/salts instead of acidic mixtures) for human safety and environmental concerns. The findings highlighted by this present review can be summarized as follows:

i. Not only does electropolishing provide a shiny and burr-free surface, but it also has the potential to shape and polish precision micro-features. Theories of electropolishing explain the difference of current density distribution at the peak and recess areas of a surface. This leads to more material removal at the peaks than at recessed areas. Consequently, the effect can be used to shape micro-scale features.
ii. Micro-fabrication processes such as μ-milling, μ-EDM, laser polish/ablation, metal-assisted chemical etching, LIGA, and reactive ion etching have been used for micro-nano-fabrication with high-aspect ratios and complex geometries. Owing to the limitations of material removal along a linear path (rectilinear nature of light, plasma, and mechanical tools), they cannot be used to shape/form micro-features.
iii. There are some important process parameters; specifically, voltage, current density, temperature, stationary/mobile electrodes, EP time, and convection, which affect material removal rate, surface roughness (Ra), and shine. The electrolytes can be characterized through techniques including linear scanning voltammetry (LSV), cyclic voltammetry (CYV), gravimetric analysis (GA), and chrono-amperometric analysis (CAA), which indicates the current limiting plateau region for electropolishing. This helps in selecting suitable process parameters to optimize a particular EP process.
iv. Researchers are increasingly motivated to use environmentally friendly electrolytes. Conventional (acid-based) electrolytes are increasingly being replaced by mixtures of salts/ionic liquids, as per the new term ‘deep eutectic solvents’ (‘DES’). Electrolytes (sulfuric/phosphoric/acetic acid) that worked well for alloys of steel, copper, aluminum, titanium, and niobium are increasingly being replaced by mixtures of choline chloride (ChCl) and ethylene glycol (EG), and other salts.
v. Achieving a micro-level surface polish is more challenging than general electropolishing, as it pertains to complex geometries, narrow channels, and special forms within miniature components. The control of material removal rates, the viscosity of electrolyte, optimum process parameters and a controlled environment are crucial for avoiding defects, removing burrs, enhancing corrosion resistance, and combining micro-level polish with structural integrity. Some of the most significant micro-scale applications have been discussed in this review; these include bio-medical stents, implants, AM parts, radio frequency-based RF MEMS, and superconducting devices, and TEM samples.
vi. Novel developments of electropolishing for shaping micro-features have been demonstrated within our own work by means of a series of micro-arrays. There is a gradient difference due to the uneven surface of the metal, which causes a higher current density to exist at peak regions than at valley areas. By using this characteristic intelligently, the micro-features can be shaped using electropolishing after other micro-fabrication methods, e.g., micro-milling, micro-electroforming, and micro-EDM. It is a versatile process for fabricating complex 3D geometries which cannot be achieved by other technologies. However, controlling the process to achieve optimal shape still needs to be studied and optimized.

## Figures and Tables

**Table 1:** Micro-feature sizes obtained from various manufacturing processes.

Micro-Fabrication Technique	Minimum Feature Size	Theoretical Draft Angle	Aspect Ratio	Process Limitations	Ref.
Micro-milling	25 μm	0–90 (3D)	No real limits	Tool deflection, vibrational forces, wear, burrs, and machining tracks.	[47]
Micro-EDM	15 μm	0–90 (3D)	14	Surface crack density, tool wear, low efficiency, and surface form less accessible by machine tool.	[79]
Laser polishing/ablation	10 μm	0–90 (3D)	2–2.5	High cost involved, surface cracks and oxidation can damage the micro-features.	[114]
LIGA	10 nm	1–10 (2.5D)	2	Substrate breakage due to delamination, flatness, non-uniformity and demolding forces.	[60]
Chemical etching	200 nm	NA	1.5	Etching induced damage, unequal etch rates and isotropic issues.	[115]
Reactive Ion etching	6 μm	NA	up to 30	Complex cost of equipment and side wall defect issues.	[110]
